# Impact of low high-density lipoprotein-cholesterol level on 2-year clinical outcomes after acute myocardial infarction in patients with diabetes mellitus

**DOI:** 10.1186/s12944-016-0374-5

**Published:** 2016-11-18

**Authors:** Hyung Joon Joo, Sang-A Cho, Soon Jun Hong, Seung-Ho Hur, Jang-Ho Bae, Dong-Ju Choi, Young-Keun Ahn, Jong-Seon Park, Rak-Kyeong Choi, Donghoon Choi, Joon-Hong Kim, Kyoo-Rok Han, Hun-Sik Park, So-Yeon Choi, Jung-Han Yoon, Hyeon-Cheol Kwon, Seung-Woon Rha, Kyung-Kuk Hwang, Kyung-Tae Jung, Seok-Kyu Oh, Jae-Hwan Lee, Eun-Seok Shin, Kee-Sik Kim, Hyo-Soo Kim, Do-Sun Lim

**Affiliations:** 1 Division of Cardiology, Korea University Anam Hospital, 126-1, 5 ka, Anam-dong, Sungbuk-ku, Seoul 136-705 Republic of Korea; 2 Division of Cardiology, Keimyung University Dongsan Medical Center, Daegu, South Korea; 3 Division of Cardiology, Konyang University Hospital, Daejeon, South Korea; 4 Division of Cardiology, Seoul National University Bundang Hospital, Seongnam, South Korea; 5 Division of Cardiology, Chonnam National University Hospital, Gwangju, South Korea; 6 Division of Cardiology, Yeungnam University Hospital, Daegu, South Korea; 7 Division of Cardiology, Sejong General Hospital, Bucheon, South Korea; 8 Division of Cardiology, Yonsei University Severance Hospital, Seoul, South Korea; 9 Division of Cardiology, Pusan National University Yangsan Hospital, Yangsan, South Korea; 10 Division of Cardiology, Hallym University Kangdong Sacred Heart Hospital, Seoul, South Korea; 11 Division of Cardiology, Kyungpook National University Hospital, Daegu, South Korea; 12 Division of Cardiology, Ajou University Hospital, Suwon, South Korea; 13 Division of Cardiology, Wonju Severance Christian Hospital, Wonju, South Korea; 14 Division of Cardiology, Samsung Medical Center, Seoul, South Korea; 15 Division of Cardiology, Korea University Guro Hospital, Seoul, South Korea; 16 Division of Cardiology, Chungbuk National University Hospital, Cheongju, South Korea; 17 Division of Cardiology, Eulji University Hospital, Daejeon, South Korea; 18 Division of Cardiology, Wonkwang University Hospital, Iksan, South Korea; 19 Division of Cardiology, Chungnam National University Hospital, Daejeon, South Korea; 20 Division of Cardiology, Ulsan University Hospital, Ulsan, South Korea; 21 Division of Cardiology, Daegu Catholic University Medical Center, Daegu, South Korea; 22 Division of Cardiology, Seoul National University Hospital, 101, DaeHak-ro, JongRo-gu, Seoul, 110-744 Republic of Korea

**Keywords:** High-density lipoprotein cholesterol, Major adverse cardiovascular events, Acute myocardial infarction, Diabetes mellitus

## Abstract

**Background:**

It is still unclear whether low high-density lipoprotein cholesterol (HDL-C) affects cardiovascular outcomes after acute myocardial infarction (AMI), especially in patients with diabetes mellitus.

**Methods:**

A total of 984 AMI patients with diabetes mellitus from the DIabetic Acute Myocardial InfarctiON Disease (DIAMOND) Korean multicenter registry were divided into two groups based on HDL-C level on admission: normal HDL-C group (HDL-C ≥ 40 mg/dL, *n* = 519) and low HDL-C group (HDL-C < 40 mg/dL, *n* = 465). The primary endpoint was 2-year major adverse cardiovascular events (MACE), defined as a composite of cardiac death, non-fatal myocardial infarction (MI), and target vessel revascularization (TVR).

**Results:**

The median follow-up duration was 730 days. The 2-year MACE rates were significantly higher in the low HDL-C group than in the normal HDL-C group (MACE, 7.44% vs. 3.49%, *p* = 0.006; cardiac death, 3.72% vs. 0.97%, *p* = 0.004; non-fatal MI, 1.75% vs. 1.55%, *p* = 0.806; TVR, 3.50% vs. 0.97%, *p* = 0.007). Kaplan-Meier analysis revealed that the low HDL-C group had a significantly higher incidence of MACE compared to the normal HDL-C group (log-rank *p* = 0.013). After adjusting for conventional risk factors, Cox proportional hazards analysis suggested that low HDL-C was an independent risk predictor for MACE (hazard ratio [HR] 3.075, 95% confidence interval [CI] 1.034-9.144, *p* = 0.043).

**Conclusions:**

In patients with diabetes mellitus, low HDL-C remained an independent risk predictor for MACE after adjusting for multiple risk factors during 2-year follow-up of AMI.

**Trial registration:**

This study was the sub-analysis of the prospective multi-center registry of DIAMOND (Diabetic acute myocardial infarction Disease) in Korea. This is the observational study supported by Bayer HealthCare, Korea. Study number is 15614. First patient first visit was 02 April 2010 and last patient last visit was 09 December 2013.

**Electronic supplementary material:**

The online version of this article (doi:10.1186/s12944-016-0374-5) contains supplementary material, which is available to authorized users.

## Background

Acute myocardial infarction (AMI) is a leading cause of mortality in patients with diabetes mellitus. Recent data revealed a 10–15% 1-year mortality rate after AMI in a diabetic population [1]. Korean data also showed a higher mortality rate after AMI in diabetic patients compared to non-diabetic patients [2]. Preventive strategies targeting platelet activity and lipid profiles in addition to glycemic control and lifestyle modification are an essential part of management in these patients [3, 4].

Previous primary prevention trials revealed that low high-density lipoprotein cholesterol (HDL-C) level is a significant risk factor for cardiovascular events in the general population [5, 6]. The Treating to New Targets (TNT) study revealed that approximately 15% of patients with diabetes mellitus have low HDL-C level [7]. In diabetes, insulin resistance increases triglyceride-enriched HDL particles and causes more rapid clearance of HDL particles [8]. Thus, low HDL-C is more common in diabetic patients. Moreover, previous epidemiologic studies demonstrated a higher prevalence of low HDL-C in the Asian population [9, 10]. The association between low HDL-C and coronary heart disease seemed to be stronger in the Asian population compared to non-Asians [11].

Recently, low HDL-C levels have been reportedly associated with a higher rate of cardiovascular events in patients with stable coronary artery disease, percutaneous coronary intervention, or even AMI [12–14]. However, it is still controversial whether low HDL-C affects cardiovascular outcomes after AMI. In addition, no studies have evaluated AMI patients with diabetes mellitus. In the present study, we have investigated the prevalence of low HDL-C and its long-term clinical impact in diabetic patients after AMI.

## Methods

### Study design

The DIAMOND (DIabetic Acute Myocardial infarctiON Disease registry in Korea) study was a multicenter, prospective observational study [15]. Briefly, between April 2010 and December 2013, 1,198 diabetic patients admitted for AMI were enrolled at 22 institutions in South Korea. The study participants were encouraged to follow up at 1, 6, 12, and 24 months after discharge. The study was approved by the institutional review board of each institute and performed in accordance with the principles of the Declaration of Helsinki. Written informed consent was obtained from all patients.

The present study was a retrospective analysis of previously collected data that were locked at December 2014. During the follow-up period, 6 patients withdrew consent, 79 never followed up after discharge, and 129 had missing values for laboratory findings on admission. Finally, 984 patients were analyzed.

### Definitions

AMI was defined based on elevated cardiac troponin-I or T level (exceeding upper limit of normal) or creatine kinase-MB fraction (CK-MB) (exceeding three times upper limit of normal), along with angiographic evidence. Angiographic evidence for AMI included significant coronary stenosis, i.e., more than 50% luminal stenosis, intracoronary filling defect or haziness suggesting coronary thrombus/vulnerable plaque, or coronary artery vasospasm confirmed by intracoronary acetylcholine or ergonovine provocation test. Diabetes mellitus was defined by fasting plasma glucose level on two separate occasions ≥ 126 mg/dL, a random plasma glucose level ≥ 200 mg/dL, 2-h plasma glucose post-75 g dextrose load on two separate occasions ≥ 200 mg/dL, or taking oral hypoglycemic agents or using insulin. Dyslipidemia was defined as total cholesterol level ≥ 240 mg/dL, low-density lipoprotein cholesterol (LDL-C) level ≥ 130 mg/dL, HDL-C level < 40 mg/dL, triglyceride level ≥ 150 mg/dL, and/or treatment with lipid lowering agents. Low HDL-C was defined as < 40 mg/dL. Renal function was estimated with the glomerular filtration rate (eGFR), which was calculated with the Modification of Diet in Renal Disease (MDRD) equation as following: eGFR (mL/min/1.73 m^2^) = 175 × (serum creatinine level)^-1.154^ × (age)^-0.203^ × (0.742 if female) [16].

### Endpoint

In the present analysis, major adverse cardiac events (MACE) was defined as a composite of cardiac death, non-fatal myocardial infarction (MI), and target vessel revascularization (TVR). Revascularization other than TVR (non-TVR) was also analyzed. Definite stent thrombosis was assessed according to the Academic Research Consortium definition.

### Statistical analysis

Categorical variables were reported as count (percentage) and continuous variables as the mean ± standard deviation. Comparisons between two groups were performed using the independent Student’s t-test for continuous variables, and the χ2 test for categorical variables. Kaplan–Meier survival curves with a log-rank test and Cox proportional hazard model analyses were performed to compare the long-term incidence of MACE and cardiac death between the two groups. The univariate and multivariate Cox proportional hazard regression analyses were used to identify risk predictors for MACE and cardiac death. The risk factors were tested with the multivariate Cox proportional hazard regression model by the backward selection method. The candidate variables for the model included HDL-C level, age, men, body mass index (BMI), current smoking, previous MI, ST-segment elevation myocardial infarction (STEMI) on admission, primary percutaneous coronary intervention (PCI), hypertension, statin use, estimated glomerular filtration rate (eGFR), hemoglobin A1c (HbA1c) level, high-sensitivity C-reactive protein (hsCRP) level, LDL-C level, left ventricular ejection fraction (LVEF), multivessel disease, lesion type (B2/C), stent diameter ≤ 2.75 mm, and stent length ≥ 28 mm. The selection significance level was 0.1. The results were expressed as the hazard ratio (HR) with a 95% confidence interval (CI) and p-value. All tests were two-tailed, and *p*-values less than 0.05 were considered statistically significant. All statistical analyses were performed using SAS (v. 9.3, SAS Institute Inc., USA).

## Results

Among a total of 984 diabetic patients who experienced AMI, 465 patients (47.3%) were in the low HDL-C group. Baseline clinical characteristics are summarized in Table 1. The low HDL-C group had more men (*p* = 0.002). There were fewer patients with newly diagnosed diabetes mellitus in the low HDL-C group (*p* = 0.034). Laboratory findings showed lower total cholesterol and higher triglyceride levels in the low HDL-C group (*p* < .001). Angiographic findings showed no significant difference between the two groups (Table 2).

**Table 1:** Baseline clinical characteristics

	Low HDL (*n* = 465)	Normal HDL (*n* = 519)	*p*-value
Age (years)	64.12 ± 9.91	65.10 ± 9.78	0.120
Male, n (%)	328 (70.54)	318 (61.27)	0.002
BMI (kg/m^2^)	24.23 ± 3.01	24.06 ± 3.02	0.301
Smoking, n (%)	164 (35.42)	166 (31.98)	0.255
Newly diagnosed DM, n (%)	30 (6.45)	53 (10.21)	0.034
Hypertension, n (%)	302 (65.09)	335 (64.92)	0.957
Dyslipidemia, n (%)	115 (24.78)	149 (28.76)	0.160
Previous MI, n (%)	28 (6.02)	33 (6.36)	0.827
On Admission			
STEMI, n (%)	217 (46.67)	254 (48.94)	0.476
Primary PCI, n (%)	280 (60.22)	318 (61.27)	0.735
LVEF, n (%)	50.51 ± 12.31	51.00 ± 11.26	0.522
Total cholesterol (mg/dL)	162.92 ± 45.74	180.83 ± 44.34	<.001
Triglyceride (mg/dL)	151.59 ± 109.69	121.63 ± 83.35	<.001
LDL-C (mg/dL)	100.97 ± 34.76	105.63 ± 45.82	0.072
HDL-C (mg/dL)	32.70 ± 5.22	53.8 ± 26.83	<.001
Creatinine (mg/dL)	2.16 ± 16.55	1.69 ± 11.84	0.611
HbA1c (%)	7.83 ± 1.58	7.66 ± 1.49	0.111
hsCRP (mg/L)	6.00 ± 15.69	6.01 ± 24.07	0.993
Peak CK-MB (ng/mL)	75.71 ± 120.75	85.67 ± 122.03	0.202
Maximum Troponin-I (ng/mL)	28.76 ± 55.53	29.71 ± 58.47	0.825
Medication at discharge			
Aspirin, n (%)	449 (98.25)	510 (98.84)	0.442
Clopidogrel, n (%)	434 (94.97)	487 (94.38)	0.684
Cilostazol, n (%)	88 (19.26)	105 (20.35)	0.670
Beta blocker, n (%)	394 (85.65)	437 (84.36)	0.573
ACEI/ARB, n (%)	376 (81.74)	441 (85.14)	0.153
Statin, n (%)	381 (82.83)	452 (87.26)	0.052
Nitrate, n (%)	128 (27.83)	149 (28.76)	0.745
Insulin, n (%)	76 (16.52)	70 (13.51)	0.188

Data are presented as mean ± SD for continuous variables and numbers (%) for categorical variables. *BMI* body mass index, *DM* diabetes mellitus, *MI* myocardial infarction, *STEMI* ST-segment elevation MI, *PCI* percutaneous coronary intervention, *LVEF* left ventricular ejection fraction, *LDL-C* low-density lipoprotein cholesterol, *HDL-C* high-density lipoprotein cholesterol, *HbA1c* hemoglobin A1c, *hsCRP* high-sensitivity C-reactive protein, *CK-MB* creatine kinase-MB, *ACEI* angiotensin-converting enzyme inhibitor, *ARB* angiotensin II receptor blocker

**Table 2:** Angiographic and procedural characteristics

	Low HDL	Normal HDL	*p*-value
Target vessel, n (%)			
Left main	14 (3.01)	11 (2.12)	0.375
LAD	224 (48.17)	270 (52.02)	0.228
LCX	114 (24.52)	144 (27.75)	0.250
RCA	175 (37.63)	178 (34.30)	0.276
Multivessel disease, n (%)	284 (61.08)	302 (58.19)	0.357
Type B2/C lesion, n (%)	371 (84.9)	403 (80.76)	0.095
TIMI grade, n (%)			
0	187 (42.79)	190 (38.08)	0.404
1	52 (11.90)	64 (12.83)	
2	46 (10.53)	78 (15.63)	
3	152 (34.78)	167 (33.47)	
Drug-eluting stent, n (%)	398 (98.76)	445 (97.8)	0.294
Stent diameter (mm)	3.10 ± 0.45	3.13 ± 0.44	0.215
Stent length (mm)	25.44 ± 8.25	24.71 ± 9.12	0.272
Stent number	1.57 ± 0.89	1.55 ± 0.82	0.722

Data are presented as mean ± SD for continuous variables and numbers (%) for categorical variables. *LAD* left anterior descending artery, *LCX* left circumflex artery, *RCA* right coronary artery

**Fig. 1:**
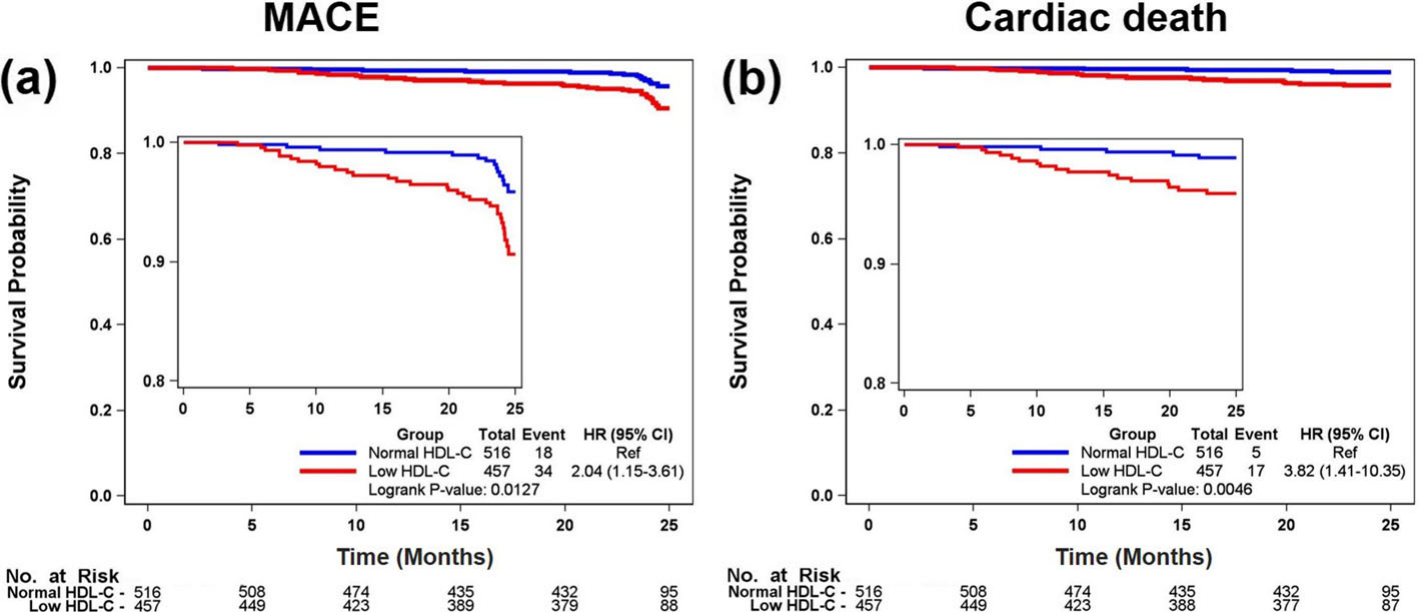
Kaplan-Meier analysis of low HDL-C and normal HDL-C groups. **a** cumulative MACE-free survival. **b** cumulative cardiac death-free survival. HR, hazard ratio; 95% CI, 95% confidence interval; Ref, reference

In-hospital and 2-year clinical outcomes are shown in Table 3. There were no significant differences in in-hospital deaths and complications between the two groups. The 2-year clinical outcomes were accessed in the remaining 973 patients after excluding the patients with in-hospital death. Median follow-up period was 730 days. During the follow-up period, the incidence of MACE, cardiac death, and TVR was significantly higher in the low HDL-C group (MACE, 7.44% vs. 3.49%, *p* = 0.006; cardiac death, 3.72% vs. 0.97%, *p* = 0.004; non-fatal MI, 1.75% vs. 1.55%, *p* = 0.806; TVR, 3.50% vs. 0.97%, *p* = 0.007). Kaplan-Meier analysis revealed that the low HDL-C group had a significantly higher incidence of MACE and cardiac death compared to the normal HDL-C group (MACE, log-rank *p* = 0.012; cardiac death, log-rank *p* = 0.005; Fig. 1).

**Table 3:** In-hospital and 2-year clinical outcomes after acute myocardial infarction

	Low HDL	Normal HDL	*p*-value
In-hospital			
Death	8 (1.72)	3 (0.58)	0.089
Cardiogenic shock	10 (2.15)	6 (1.16)	0.218
Acute renal failure	5 (1.08)	2 (0.39)	0.199
Major bleeding	4 (0.86)	6 (1.16)	0.644
During follow-up period			
MACE	34 (7.44)	18 (3.49)	0.006
Cardiac death	17 (3.72)	5 (0.97)	0.004
Non-fatal MI	8 (1.75)	8 (1.55)	0.806
TVR	16 (3.50)	5 (0.97)	0.007
Non-TVR	11 (2.41)	12 (2.33)	0.934
Stent thrombosis, definite	3 (0.65)	1 (0.19)	0.266

Data are presented as numbers (%) for categorical variables. *MACE* major adverse cardiac event, *MI* myocardial infarction, *TVR* target vessel revascularization

In multivariable Cox proportional hazard model analyses, HDL-C level, BMI, hypertension, and eGFR were independent significant predictors for MACE [HDL-C, HR (95% CI) 0.95 (0.905 - 0.999), *p* = 0. 047; BMI, HR (95% CI) 0.84 (0.714 – 0.993), *p* = 0.041; hypertension, HR (95% CI) 4.80 (1.052 – 21.927), *p* = 0.043; eGFR, HR (95% CI) 0.981 (0.966 – 0.996), *p* = 0.016] after adjusting for conventional risk factors (Table 4). LVEF remained the only independent predictor for cardiac death [HR (95% CI) 0.893 (0.828 – 0.964), *p* = 0.004].

**Table 4:** Univariate and multivariate analysis for risk factors to predict MACE and cardiac death

	MACE			Cardiac death		
Risk Factor	*β*	HR (95% CI)	*p*-value	*β*	HR (95% CI)	*p*-value
Univariate analysis						
Age	0.04	1.04 (1.007 – 1.066)	0.014	0.10	1.10 (1.048 – 1.158)	<0.001
Male	−0.10	0.90 (0.513 – 1.588)	0.723	−0.33	0.72 (0.306 – 1.677)	0.443
BMI	−0.02	0.98 (0.895 – 1.073)	0.666	−0.20	0.82 (0.703 – 0.947)	0.008
Current Smoking	−0.41	0.66 (0.354 – 1.243)	0.200	−0.56	0.57 (0.210 – 1.543)	0.268
Previous MI	−0.55	0.58 (0.327 – 1.026)	0.061	−0.92	0.40 (0.157 – 1.023)	0.056
Hypertension	1.67	5.31 (2.113 – 13.363)	<0.001	1.26	3.51 (1.038 – 11.858)	0.043
HDL-C	−0.05	0.95 (0.928 – 0.981)	0.001	−0.08	0.92 (0.879 – 0.963)	<0.001
LDL-C	−0.01	0.99 (0.986 – 1.001)	0.098	−0.01	1.00 (0.983 – 1.007)	0.395
eGFR	−0.02	0.99 (0.977 – 0.994)	0.001	−0.03	0.973 (0.960 – 0.986)	<0.001
Hba1c	0.02	1.02 (0.852 – 1.231)	0.802	0.13	1.14 (0.880 – 1.482)	0.319
hsCRP	0.002	1.00 (0.988 – 1.017)	0.786	0.003	1.00 (0.985 – 1.022)	0.739
STEMI at admission	−0.55	0.58 (0.327 – 1.026)	0.061	−0.92	0.40 (0.157 – 1.023)	0.056
Primary PCI	−0.38	0.685 (0.397 – 1.183)	0.175	−1.27	0.28 (0.115 – 0.693)	0.006
MVD	0.13	1.14 (0.649 – 2.008)	0.647	−0.22	0.80 (0.347 – 1.859)	0.608
Lesion type (B2/C)	0.05	1.05 (0.466 – 2.346)	0.914	−0.60	0.55 (0.175 – 1.727)	0.306
Stent diameter ≤2.75 mm	0.02	1.02 (0.490 – 2.124)	0.958	−1.27	0.28 (0.035 - 2.211)	0.227
Stent length ≥28 mm	0.49	1.64 (0.842 – 3.174)	0.146	0.41	1.51 (0.437 – 5.209)	0.516
LVEF	−0.04	0.96 (0.941 – 0.986)	0.002	−0.10	0.904 (0.870 – 0.939)	<0.001
Statin at discharge	−0.35	0.71 (0.354 – 1.407)	0.322	−0.83	0.44 (0.170 – 1.113)	0.083
Multivariate analysis						
HDL-C	−0.05	0.95 (0.905 - 0.999)	0.047	-	-	-
Age	−0.05	0.96 (0.906 – 1.006)	0.085	-	-	-
BMI	−0.17	0.84 (0.714 – 0.993)	0.041	−0.31	0.73 (0.537 – 1.002)	0.051
Hypertension	1.57	4.80 (1.052 – 21.927)	0.043	-	-	-
eGFR	−0.02	0.981 (0.966 – 0.996)	0.016	−0.02	0.98 (0.954-1.004)	0.093
Stent diameter ≤2.75 mm	-	-	-	−1.81	0.16 (0.017 – 1.587)	0.119
LVEF	-	-	-	−0.11	0.893 (0.828 – 0.964)	0.004

*MACE* major adverse cardiovascular events, *HR* hazard ratio, *95% CI* 95% confidence interval, *BMI* body mass index, *MI* myocardial infarction, *LDL-C* low-density lipoprotein cholesterol, *eGFR* estimated glomerular filtration rate, *hsCRP* high-sensitivity C-reactive protein, *STEMI* ST-segment elevation myocardial infarction, *PCI* percutaneous coronary intervention, *MVD* multi-vessel disease, *LVEF* left ventricular ejection fraction, *n.d.* not determined

**Fig. 2:**
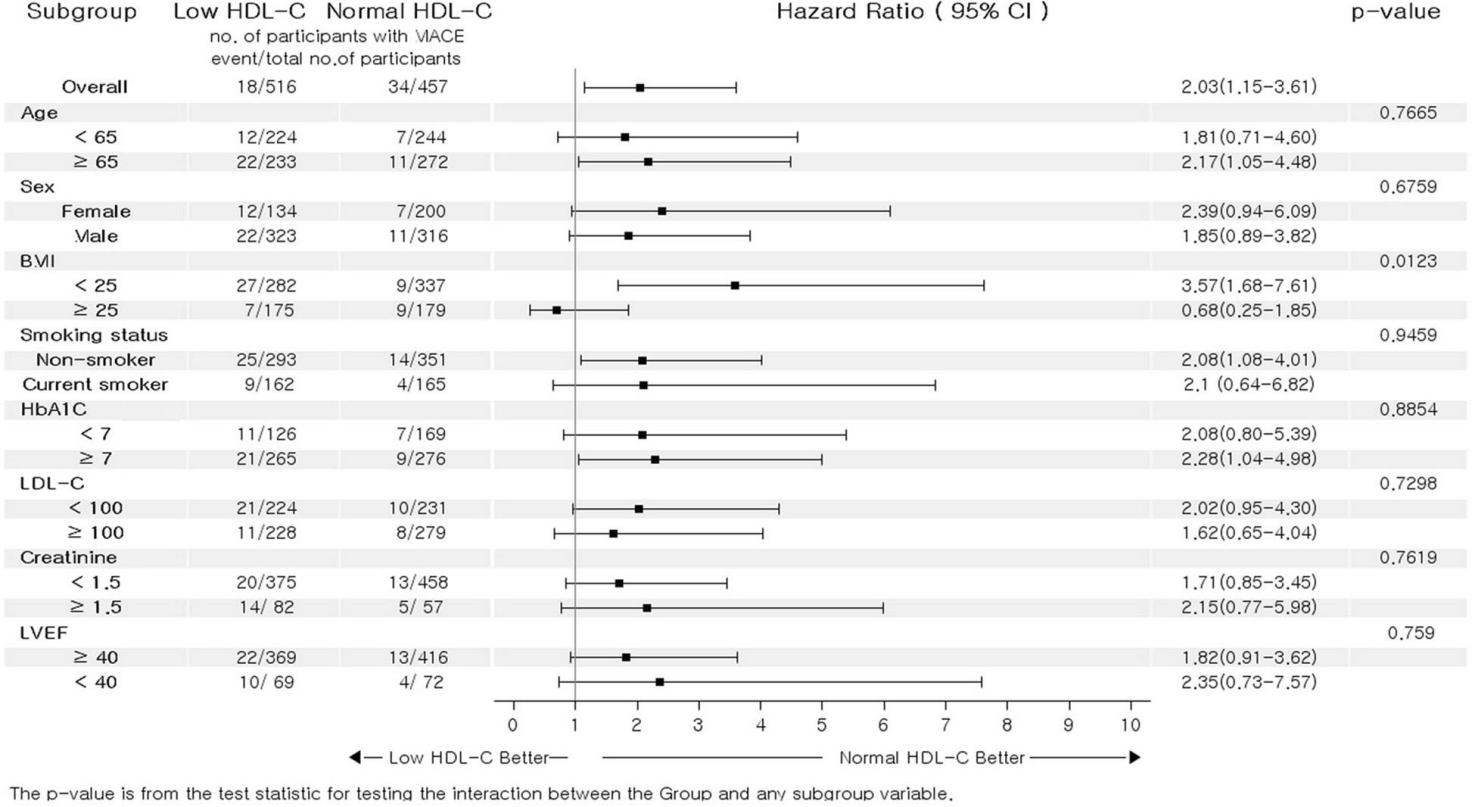
Comparative unadjusted hazard ratios of MACE for subgroups. MACE, major adverse cardiovascular events; 95% CI, 95% confidence interval; BMI, body mass index; LDL-C, low-density lipoprotein cholesterol; LVEF, left ventricular ejection fraction

Next, the unadjusted HRs for MACE were calculated in various subgroups based on age, sex, BMI, smoking, HbA1c, LDL-C, creatinine, and LVEF (Fig. 2). Interestingly, statistical significance was found in patients with high BMI. There were no significant interactions between HDL-C and MACE among the other 7 subgroups.

## Discussion

The main findings of the present study are as follows: (1) 46.2% of diabetic patients presenting with AMI had a low HDL-C level; (2) 2-year clinical outcomes including MACE (mainly cardiac death and TVR) were poorer in diabetic patients with a low HDL-C level after AMI compared to those with a normal HDL-C level; (3) low HDL-C level remained an important risk predictor for MACE after adjusting for confounding clinical factors.

Previous community-based primary prevention studies showed that low HDL-cholesterol level was strongly associated with poor cardiovascular outcome in the general population [17, 18]. Current guidelines strongly recommend statin therapy for patients with overt atherosclerotic vascular diseases and diabetes mellitus [19, 20]. A previous study demonstrated that statin therapy increased HDL-C level by approximately 7.5%, and was associated with coronary atherosclerotic regression [21]. However, more than 40% of statin-treated patients have a persistently low HDL-C level [22, 23]. Several studies also suggested low HDL-C as an independent risk predictor, even in patients with overt atherosclerotic vascular diseases on statin therapy. Seo et al. reported that a low HDL-C level on statin therapy was associated with poor clinical outcome after PCI [12]. Ogital et al. also showed that low HDL-C was a risk factor in diabetic patients with stable coronary artery disease [13]. Recently, Lee et al. showed similar results in patients with AMI [14]. The present study showed a higher MACE rate in diabetic AMI patients with low HDL-C level compared to those with a normal HDL-C level.

On the other hand, several studies have questioned the impact of HDL-C on cardiovascular prognosis. Izuhara et al. showed that the statistical significance of low HDL-C in poor clinical outcomes disappeared after adjusting for confounding factors in patients who underwent PCI [23]. Angeloni et al. showed similar 3-year MACE rates in low and high HDL-C groups, even in patients who underwent coronary artery bypass grafting [24]. Ji et al. also showed no significant difference in 1-year MACE rates between the two groups in AMI patients [25].

The discrepancy among studies might be explained by several factors. First, the studies were performed in different clinical settings and had different demographic and risk profiles. The clinical situations could have affected the anti-atherogenic and anti-inflammatory function of HDL-C. Recently, many studies have focused on the function of HDL-C rather than the level. HDL-C plays an important role in atherogenesis through reverse cholesterol transport. Removing cholesterol from macrophages (called “macrophage cholesterol efflux”) is significantly associated with cardiovascular events [26, 27]. Cholesterol efflux capacity and the NO-producing effect of HDL-C were also decreased in patients with acute coronary syndrome [28, 29]. Dysfunction of HDL-C was also reported in diabetic patients [30]. These findings suggested that HDL-C dysfunction might mask the clinical significance of serum HDL-C level for cardiovascular prognosis depending on the clinical situation. In other words, the quality of HDL-C might be more significant than the quantity in selected populations. Second, the cut-off value of HDL-C could affect the results of clinical studies. Interestingly, the studies using the cut-off value of 40 mg/dL suggested that low HDL-C was an independent risk predictor [12–14]. Other studies using different cut-off values for men and women (40 mg/dL for men and 50 mg/dL for women) failed to show the significance of low HDL-C [23–25]. More importantly, 2 studies from the same AMI registry showed different results. One adopted the cut-off value of 40 mg/dL for both men and women [14], and the other study used different cut-off values for men and women (40 mg/dL for men and 50 mg/dL for women) [25]. In the present study, receiver operating characteristic (ROC) curves of HDL-C for cardiac death showed that the area under the curve (AUC) for men was 0.722 and 0.753 for women (Additional file 1: Figure S1); optimal cut-off points with the Youden index were 38 mg/dL for men and 35 mg/dL for women. ROC curves of HDL-C for MACE showed that the AUC for men was 0.634 and 0.660 for women; optimal cut-off points with the Youden index were 38 mg/dL for men and 40 mg/dL for women. Thus, we used the same cut-off value of 40 mg/dL for both men and women. Moreover, 2015 Korean guidelines for the management of dyslipidemia adopted a criterion of below 40 mg/dL as low HDL-C for both men and women [31].

A genetic mechanism reportedly links low HDL-C and inflammatory states [32]. Hoven et al. also showed a clinical relationship between low HDL-C level and its inflammatory and oxidative phenotype [33]. Moreover, there is much experimental evidence for the beneficial effects of HDL-C [34]. Although previous clinical trials aimed at raising HDL-C failed to show promising results [35–38], new HDL-C-based strategies designed to improve HDL-C functionality instead of increasing the HDL-C level have been under development [39, 40].

There are several limitations. First, the study subjects were divided into only 2 groups. We did not address the impact of the other ranges of HDL-C level (e.g., HDL-C > 70 mg/dL or < 20 mg/dL) due to the limited patient numbers. Thus, the possible protective role of high HDL-C level or its dose–response relationship could not be investigated. Second, the current guidelines recommend statin therapy for diabetic patients regardless of their lipid profile [31, 41]. Detailed information (name and dose) on statins and other medications affecting HDL-C levels were not assessed. However, the effect of statins on HDL-C has been known to be relatively small. Moreover, our data highlighted the clinical limitations of current statin usage and proposed HDL-C as a therapeutic target despite the failures of previous trials. Third, the follow-up rate of HDL-C was only 62.0% in the present study. Data on HDL-C levels before admission were not obtained. Thus, we cannot analyze the dynamics of HDL-C. Fourth, serum uric acid level was not included and adjusted for a potential confounding factor. Although the relationship between serum uric acid level and the prognosis of acute myocardial infarction has been still controversial, serum uric acid level is a well-known surrogate marker for inflammation and atherosclerosis [42]. Unfortunately, serum uric acid level was not available in our registry. Additional data including serum uric acid level and other inflammatory biomarkers could be more informative to understanding the clinical impact of HDL-C.

## Conclusions

The 2-year incidence of MACE, cardiac death, and TVR was significantly higher in diabetic patients with a low HDL-C level compared to those with a normal HDL-C level after AMI. Low HDL-C level remained an independent risk predictor for both MACE and cardiac death after adjusting for multiple risk factors.

## Additional file

Additional file 1: Figure S1. Receiver operating characteristic (ROC) curves of HDL-C for MACE and cardiac death. (DOCX 346 kb)

## Abbreviations

AMI: Acute myocardial infarction
AUC: Area under the curve
BMI: Body mass index
CI: Confidence interval
CKD: Chronic kidney disease
CK-MB: Creatine kinase-MB fraction
DIAMOND: DIabetic acute myocardial InfarctiON disease
HbA1c: Hemoglobin A1c
HDL-C: High-density lipoprotein cholesterol
HR: Hazard ratio
hsCRP: High-sensitivity C-reactive protein
LDL-C: Low-density lipoprotein cholesterol
LVEF: Left ventricular ejection fraction
MACE: Major adverse cardiovascular events
MI: Myocardial infarction
PCI: Percutaneous coronary intervention
ROC: Receiver operating characteristic
STEMI: ST-segment elevation myocardial infarction
TNT: Treating to new targets
TVR: Target vessel revascularization
