# Supplementary material for: Impact of low high-density lipoprotein-cholesterol level on 2-year clinical outcomes after acute myocardial infarction in patients with diabetes mellitus
Source: Lipids Health Dis. 2016 Nov 18;15:197. doi: 10.1186/s12944-016-0374-5 (PMC5116186; doi:10.1186/s12944-016-0374-5)
Supplement: Additional file 1: Figure S1. — Receiver operating characteristic (ROC) curves of HDL-C for MACE and cardiac death. (DOCX 346 kb) [file 12944_2016_374_MOESM1_ESM.docx]

**SUPPLEMENTAL FIGURE**

**Supplemental Figure 1. Receiver operating characteristic (ROC) curves of HDL-C for MACE and cardiac death.**

**
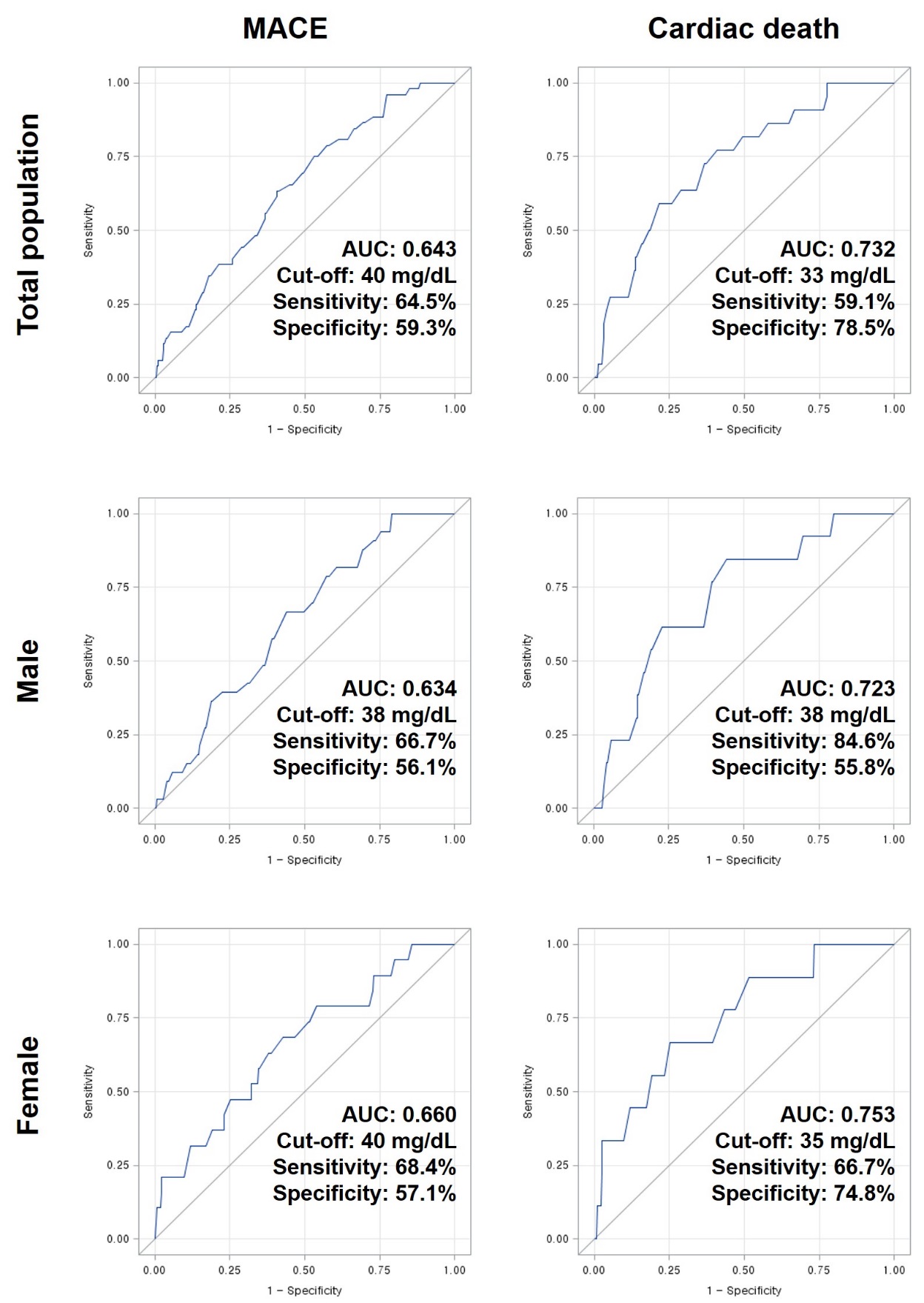
**

The cut-off points were calculated by the Youden index. AUC, area under the curve
